# Bioassay of 2, 4, 5-trichlorophenoxyacetic acid for carcinogenicity in mice.

**DOI:** 10.1038/bjc.1976.100

**Published:** 1976-06

**Authors:** I. Muranyi-Kovacs, G. Rudali, J. Imbert

## Abstract

Adult mice of strains C3Hf and XVII/G received 2, 4, 5-T by continuous oral administration (80 ppm in the diet). The 2, 4, 5-T preparation contained less than 0-05 ppm of dioxins. In 2, 4, 5-T -treated C3Hf mice a significant increase in the incidence of neoplastic lesions was found. No significant difference was found in the XVII/G strain between the treated and control mice. Rare forms of tumours, which were not observed in the controls, were present in the 2, 4, 5-t -treated C3Hf mice.


*Br. J. Cancer* (1976) **33**, 626

### BIOASSAY OF 2,4,5-TRICHLOROPHENOXYACETIC ACID FOR CARCINOGENICITY IN MICE

I. MURANYI-KOVACS*, G. RUDALI AND J. IMBERT*

*From the Laboratoire de Génétique, Fondation Curie, Institut du Radium, 26, rue d'Ulm, Paris* 75005 (*France*)

Received 12 May 1975 Accepted 4 February 1976

**Summary.—Adult mice of strains C3Hf and XVII/G received 2,4,5-T by continuous oral administration (80 ppm in the diet). The 2,4,5-T preparation contained less than 0·05 ppm of dioxins. In 2,4,5-T-treated C3Hf mice a significant increase in the incidence of neoplastic lesions was found. No significant difference was found in the XVII/G strain between the treated and control mice. Rare forms of tumours, which were not observed in the controls, were present in the 2,4,5-T-treated C3Hf mice.**

2,4,5-TRICHLOROPHENOXYACETIC ACID has been widely used as a herbicide in the U.S.A., Europe, and in massive quantities in Vietnam. Many studies on its toxicity (Grigsby and Farwell, 1950; Drill and Hiratzka, 1953; Rowe and Hymas, 1954) and metabolism (Piper *et al.*, 1973; Gehring *et al.*, 1973) have been performed. Courtney *et al.* (1970) showed that it was teratogenic for mice, but such an action was not confirmed in rats and rabbits (Emerson *et al.*, 1971; Sparschu *et al.*, 1971). Innes *et al.* (1969) reported in a preliminary communication of an extensive study on tumorigenicity of pesticides that it had no significant carcinogenic activity.

However, ocular and nervous system lesions, spontaneous abortions, trisomy 21, and other congenital malformations have been observed in inhabitants of regions of Vietnam where 2,4,5-T had been employed on a large scale. A recent report concerning these cases suggested the responsibility of 2,4,5-T-based defoliants (Ton That Tung, 1973).

In the last few years, numerous studies (Tomatis and Mohr, 1973) seem to have confirmed that a close correlation exists between mutagenic, embryotoxic and teratogenic effects on the one hand, and carcinogenic action on the other. Moreover, the carcinogenic action of some compounds can vary according to whether they are administered to foetal (transplacentally), neonatal or adult animals (Tomatis and Mohr, 1973; Toth, 1968).

In this study, we tried to determine the carcinogenic effect of 2,4,5-T in mice by continuous oral administration.

### MATERIAL AND METHODS

*2,4,5-Trichlorophenoxyacetic acid.* — This chemical was synthesized and kindly supplied by Dr Saint-Ruff. Two separate analyses for impurities kindly performed by Dr V. K. Rowe revealed that our preparation contained less than 0·05 ppm of dioxins†.

* Present address to which reprint requests should be sent: Unité 119 Inserm 27, Bd Leï Roure—13009, Marseille, France.

† Two separate analyses for impurities revealed the following data:

| | | |
|---|---|---|
| 2,3,7,8-Tetrachlorodibenzo-p-dioxin | 0·03 ppm | 0·02 ppm |
| Hexachlorodibenzo-p-dioxins | n.d. (0·01 ppm)‡ | n.d. (0·01 ppm)‡ |
| 2,4,5-Trichlorophenol | 0·9% | 0·9% |
| 2,5-Dichlorophenoxyacetic acid | 0·1% | 0·1% |
| 4,5-Dichloro-2-methoxyphenoxyacetic acid | 1·1% | 1·2% |
| 2,5-Dichloro-4-methoxyphenoxyacetic acid, 2,4-Dichloro-5-methoxyphenoxyacetic acid | 3·9% | 4·2% |

‡ n.d. = Not detected at limit of detection indicated.

Dioxins were determined by Gas Diffusion–Mass Spectogram after extraction of an alkaline solution of a 20 mg sample with hexane and cleanup by liquid chromatography on alumina. The other analyses were conducted using methylation followed by gas chromatography and flame ionization detector and comparison with known standards.

*Administration of 2,4,5-T*.—For 2 months, beginning at 6 weeks of age, XVII/G and C3Hf mice were given 2,4,5-T (100 mg/l) in their drinking water. From then until death, 2,4,5-T was mixed directly with the diet at a concentration of 80 ppm.

*Mice*.—Inbred XVII/G and C3Hf strains of mice bred in our laboratories were used. After weaning at 4 weeks of age, males and females were separated, housed 7 to a cage, and distributed in different groups. The mice were given a sterile, commercial diet (UAR 113b) and tap water *ad libitum*. The animals were examined weekly for their general state and presence of tumour and were allowed to die or sacrificed *in extremis*. Complete autopsies were performed and macroscopically altered organs fixed in Bouin's fluid containing mercuric chloride. Distension of urinary bladder with fixative was performed on sacrificed mice suspected of having a hyperplastic bladder.

*Statistical analysis*.—The means of survival times were compared by Student's "*t*" test. The numbers of tumours and/or leukaemia in each treated group and in the corresponding group of controls were compared by the method proposed by Peto (1974). In each group, results concerning males and females were tested separately (i.e. treated males were compared with control males and treated females with control females).

The tumour-bearing mice were classified into two categories according to the tumour: (1) Incidental tumours—discovered at necropsy of an animal which died from some other cause. (2) Non-incidental tumours—diagnosed during life or causing the death of the animal.

For each category of tumours thus defined, the ratio a/b was expressed for each experimental group in each 2-month period.

For the incidental tumours, the ratio a/b is given by b: the number of necropsies of animals which did not have a tumour diagnosed before death and which did not die of a tumour, and a: the number of necropsies at which tumours were found.

For the non-incidental tumours, the ratio a/b is given by b: the number of animals still alive and without diagnosed tumours at the first week of the two-month period, and a: the number of these animals dying of tumours or having a tumour diagnosed during the period.

From these tables the expected number of tumours was calculated for each period assuming that 2,4,5-T treatment and the control had the same tumour incidence. For each group the numbers calculated were added together and compared with the number of tumours (or leukaemia) actually observed in the group by the $\chi^2$ test.

### RESULTS

Table I summarizes the data concerning the survival times, the total tumour frequencies and the number of different types of tumorous lesions.

In the XVII/G strain the average survival time was significantly higher ($P < 0{\cdot}01$) in treated females, as compared with controls, whereas in C3Hf mice there was a significant ($P < 0{\cdot}001$) decrease in average survival time of the treated males, and a non-significant decrease in female survival time ($0{\cdot}05 < P < 0{\cdot}1$). In each group we tested (Student's "*t*" test) whether there was a significant difference between the average survival time of tumour-bearing animals and that of animals without tumours. A significant difference was found only in the treated XVII/G males.

The lung tumours were alveologenic pulmonary tumours which occur spontaneously in high incidence in XVII/G mice. Histologically, they could be diagnosed variously as adenoma or adenocarcinoma. No differences were found in the proportions of these two histological types in treated mice compared with controls.

Hepatomata occur spontaneously in high frequencies in C3Hf mice and especially in old males. No differences were

TABLE I.—*Incidence of Tumours and Leukaemia in 2,4,5-T-treated Mice*

| Strain | Group | | No. of mice* | Survival time (days) Mean±SEM | Animals with tumours and/or leukaemia No. | % | Survival time (days) Mean±SEM | Types of tumours or leukaemia: Lung tumour No. | Hepatoma No. | Leukaemia No. | Other tumours | Total No |
|---|---|---|---|---|---|---|---|---|---|---|---|---|
| XVII/G | Control | ♀ | 40 | 553±16 | 21 | 53 | 569±20 | 20 | — | 2 | 2 Haemangiomata | 24 |
| | | ♂ | 32 | 516±10 | 25 | 78 | 521±11 | 22 | 4 | — | 1 Forestomach tumour | 27 |
| | 2,4,5-T (80 ppm in diet) | ♀ | 19 | 632±14 | 16 | 84 | 641±15 | 15 | — | 1 | — | 16 |
| | | ♂ | 20 | 555±19 | 15 | 75 | 583±16 | 14 | — | 1 | 1 bladder papilloma (2 hyperplastic bladders)† | 16+2† |
| C3Hf | Control | ♀ | 44 | 661±12 | 9 | 21 | 680±11 | 5 | 3 | 1 | — | 9 |
| | | ♂ | 43 | 641±12 | 21 | 49 | 630±19 | 2 | 19 | — | 1 pleomorphic salivary gland tumour | 22 |
| | 2,4,5-T (80 ppm in diet) | ♀ | 25 | 621±20 | 12 | 48 | 620±32 | — | 4 | 3 | 1 osteogenic osteosarcoma<br>2 sarcomata<br>2 cutaneous tumours<br>1 tumour of cervix | 13 |
| | | ♂ | 22 | 523±23 | 12 | 55 | 511±29 | — | 10 | 2 | 1 fibrosarcoma<br>(1 hyperplastic bladder<br>1 hyperplastic forestomach)† | 13+2† |

* Effective number of mice: mice surviving > 300 days or having a tumour before 300 days of age.

† Hyperplastic bladder and forestomach not considered as tumorous.

found in the frequencies of these tumours between treated and control mice nor in the frequencies of leukaemia.

The presence of rare forms of tumour which were not seen in the control animals were noted in the treated C3Hf mice. For example, 2 cutaneous tumours, a sebaceous squamous cell carcinoma and a squamous cell carcinoma, and an osteogenic tumour with a pulmonary metastasis were seen.

A few hyperplastic lesions and a papilloma were also found in the bladders of 2,4,5-T-treated mice. In these strains inflammatory lesions of the bladder, sometimes with urinary retention, are frequent, particularly in old males. Bladder stones have been observed only exceptionally. Macroscopic bladder papillomata have never been observed in our mouse colony over the last 20 years.

Table II presents the time distribution of the incidental tumours. These tumours were essentially lung tumours and hepatomata.

Table III shows the distribution of the non-incidental tumours. These tumours were essentially leukaemia, cutaneous

TABLE II.—*Incidence of "Incidental" Tumours in 2,4,5-T-treated Mice*

| | | | Periods | | | | | | | | |
|---|---|---|---|---|---|---|---|---|---|---|---|
| Strain | Group | | 11th–12th month | 13th–14th month | 15th–16th month | 17th–18th month | 19th–20th month | 21st–22nd month | 23rd–24th month | 25th–26th month | 27th–28th month |
| XVII/G | Control | ♀ | 1/3 | — | 3/4 | 3/8 | 6/13 | 4/7 | 1/1 | 1/2 | — |
| | | ♂ | — | 1/1 | 4/7 | 10/12 | 10/12 | — | — | — | — |
| | 2,4,5-T-treated (80 ppm in diet) | ♀ | — | — | — | 1/1 | 1/4 | 10/10 | 2/2 | 2/2 | — |
| | | ♂ | 0/1 | — | 0/2 | 3/4 | 6/6 | 4/5 | 1/1 | — | — |
| C3Hf | Control | ♀ | — | — | 0/2 | 0/2 | 0/5 | 2/4 | 4/25 | 0/2 | 0/1 |
| | | ♂ | — | — | 0/1 | 5/6 | 1/4 | 3/6 | 10/24 | — | — |
| | 2,4,5-T-treated (80 ppm in diet) | ♀ | — | — | 1/2 | 1/3 | 0/1 | 0/4 | 1/4 | 1/3 | — |
| | | ♂ | 1/2 | 1/2 | 0/1 | 0/1 | 0/2 | 0/3 | 2/3 | — | — |

Key: a/b: b = No. of necropsies of animals which did not have a tumour diagnosed before death and which did not die of a tumour.
a = No. of these necropsies at which a tumour was found.

TABLE III.—*Number of "Non-incidental" Tumours and/or Leukaemia Diagnosed during Life or Causing Death in 2,4,5-T-treated Mice*

| | | | Periods | | | | | | | | |
|---|---|---|---|---|---|---|---|---|---|---|---|
| Strain | Group | | 11th–12th month | 13th–14th month | 15th–16th month | 17th–18th month | 19th–20th month | 21st–22nd month | 23rd–24th month | 25th–26th month | 27th–28th month |
| XVII/G | Control | ♀ | 0/40 | 0/37 | 0/37 | 0/33 | **1/25** | **1/11** | 0/5 | 0/2 | — |
| | | ♂ | 0/32 | 0/32 | 0/31 | 0/24 | 0/12 | — | — | — | — |
| | 2,4,5-T-treated (80 ppm in diet) | ♀ | 0/18 | 0/18 | 0/18 | 0/18 | 0/17 | 0/13 | 0/3 | 0/1 | — |
| | | ♂ | 0/20 | 0/19 | **1/19** | 0/16 | 0/12 | 0/6 | 0/1 | — | — |
| C3Hf | Control | ♀ | 0/44 | 0/44 | 0/44 | 0/42 | 0/40 | **1/35** | **2/30** | 0/3 | 0/1 |
| | | ♂ | 0/43 | 0/43 | 0/43 | 0/42 | **1/36** | **1/31** | 0/24 | — | — |
| | 2,4,5-T-treated (80 ppm in diet) | ♀ | 0/25 | **1/25** | 0/24 | 0/22 | **3/19** | **1/16** | **2/12** | **1/4** | — |
| | | ♂ | 0/22 | **2/20** | **3/18** | **2/14** | **1/9** | 0/7 | 0/3 | — | — |

Key: a/b: b = No. of animals still alive and without a diagnosed tumour at the beginning of first week of period.
a = No. of these animals dying of tumour diagnosed during the period.

TABLE IV.—*Sums for All Periods Together of Observed and Expected Number of Tumours. 2,4,5-T-treated and Control Mice*

| | | I Incidental tumours | | | | | II Non-incidental tumours | | | | | III Total yield of tumours (I + II) | | | | |
|---|---|---|---|---|---|---|---|---|---|---|---|---|---|---|---|---|
| | | Control | | 2,4,5-T | | | Control | | 2,4,5-T | | | Control | | 2,4,5-T | | |
| Strain | Group | Obs. | Exp. | Obs. | Exp. | *P* | Obs. | Exp. | Obs. | Exp. | *P* | Obs. | Exp. | Obs. | Exp. | *P* |
| XVII/G | ♀ | 19 | 21·2 | 16 | 13·8 | NS* | 2 | 1 | 0 | 1 | NS | 21 | 22·2 | 16 | 14·8 | NS |
| | ♂ | 25 | 24·5 | 14 | 14·5 | NS | 0 | 0·6 | 1 | 0·4 | NS | 25 | 25·1 | 15 | 14·9 | NS |
| | Both | 44 | 45·7 | 30 | 28·3 | NS | 2 | 1·6 | 1 | 1·4 | NS | 46 | 47·3 | 31 | 29·7 | NS |
| C3Hf | ♀ | 6 | 6·6 | 4 | 3·4 | NS | 3 | 7·3 | 8 | 3·7 | <0·02† | 9 | 13·9 | 12 | 7·1 | <0·03 |
| | ♂ | 19 | 17·6 | 4 | 5·4 | NS | 2 | 7·4 | 8 | 2·6 | <0·001† | 21 | 25 | 12 | 8 | NS |
| | Both | 25 | 24·2 | 8 | 8·8 | NS | 5 | 14·7 | 16 | 6·3 | <0·001 | 30 | 38·9 | 24 | 15·1 | <0·01 |

* NS = non-significant $P > 0{\cdot}05$.

† $\chi^2$ calculated with Yate's correction.

tumours, sarcomata of various types and a few hepatomata diagnosed during the life of the mice.

Table IV summarizes the results of the analysis of the data given in Tables II and III.

In the XVII/G mice, there was no significant difference between the number of tumour-bearing mice observed and the number expected.

In the C3Hf mice, the differences between the number of non-incidental tumours observed and expected were significant both in males and in females, whereas for incidental tumours the differences were not significant.

Over the total yield of tumours, there was a significant difference in females, whereas in the males this difference was not significant ($P \sim 0{\cdot}1$).

In this table, the results obtained by separate analysis of the data for the females and males show that there was no sex-related difference in the carcinogenic response to 2,4,5-T treatment. Thus, we could sum the results obtained for the two sexes in each group. For these sums, a significant difference appears between the observed and expected number of tumours, both in the non-incidental tumours and in total yield of tumours in C3Hf mice.

### DISCUSSION

Essentially, two reports stimulated us to undertake this work. One by Courtney *et al.* (1970) showed that 2,4,5-T is teratogenic in mice. The other, by Vietnamese doctors, invoked 2,4,5-T as the causal agent of spontaneous abortions and congenital malformations in the inhabitants of regions of large-scale application of 2,4,5-T-based defoliants.

It should be noted, however, that 2,4,5-T preparations often contain dioxin contaminants. Now 2,3,7,8-tetrachloro-dibenzo-p-dioxin has a high toxicity in adult animals (Schwetz *et al.*, 1973) and induces severe lesions in the liver, thymus and other organs in the rat, mouse and guinea-pig and also causes anomalies in mouse foetuses (Moore *et al.*, 1973). This fact suggested that the carcinogenic agent in 2,4,5-T could be the contaminating dioxins (Ton That Tung, 1973).

The preparation used by Courtney contained 30 ppm dioxins and the defoliant mixture employed in Vietnam also contained a proportion of dioxins. In other studies, such as that of Innes *et al.* (1969) and that of Fahrig (1974) in which he showed that 2,4,5-T was not mutagenic, dioxin content was not specified.

For our experiments, we used a 2,4,5-T preparation low in dioxins. The average daily oral dose of 2,4,5-T was estimated as approximately 12 mg/kg of body weight. Considering that the 2,4,5-T preparation we used contained 0·05 ppm dioxins, the dioxin dose was thus less than 1 ng/kg body weight, i.e. $5 \times 10^4$ times lower than the $LD_{50}$ for mice (50 $\mu$g) administered in a single dose (Schwetz *et al.*, 1973).

The results shown in Table I indicate that 2,4,5-T did not reduce the survival time by a generalized toxic effect. In fact, compared with the corresponding group of controls, the average survival time of the treated animals was significantly higher in the XVII/G females, not significantly different in XVII/G males and C3Hf females, and significantly lower in the C3Hf males. In view of these facts we feel that the carcinogenesis observed in our experiments should be attributed to 2,4,5-T *per se.*

Nevertheless, a problem in assessing the significance of this effect was the choice of statistical analysis. Since the average survival time was different in some experimental groups, the use of Peto's method of statistical analysis was adopted as it takes into account differences in distribution of the survival times. In particular, the frequency of the late-appearing incidental tumours is considered in respect of the number of long-surviving animals in each group.

In the treated XVII/G females the absence of a significant difference between the number of observed and expected tumour-bearing mice seems to indicate that the increase in total tumour frequency (Table I) in treated females is related to the increase in the average survival time.

On the contrary, in the treated C3Hf females the decrease in survival time, which might have been expected to lead to a decrease in tumour frequency, was accompanied by an *increase* in tumour frequency. In this strain there was a significant difference between the number of expected and observed tumours both in the females and in the males and females considered together (Table IV). This difference was due to the increase in the number of non-incidental tumours whereas there was no significant difference for incidental tumours in treated animals. The absence of a significant difference ($P \sim 0{\cdot}1$) for the total crop of tumours in the males was due to the much greater number of incidental tumours than non-incidental tumours in the controls.

In addition, half of the non-incidental tumours appeared in the first 18 months of life of the treated C3Hf mice. In the controls the first non-incidental tumours appeared after 18 months of age. This early appearance of non-incidental tumours in treated animals shows too that 2,4,5-T has carcinogenic activity in mice.

However, the choice of the experimental animal in assessment of carcinogenic potential is very important. For practical reasons rodents, particularly mice, are often used without scientific justification for such a choice. The problem of species specificity in the metabolism of chemical carcinogens has been recently stressed by Conney and Levin (1974).

The work by Gehring *et al.* (1973) on 2,4,5-T showed that the kinetics of excretion of 2,4,5-T was extremely variable from one species to another. The half-life of 2,4,5-T in the plasma after a dose of 5 mg/kg was found to be 4·7 h in the rat, 77 h in the dog and 23 h in man. So the mouse may not be the ideal experimental model for testing the carcinogenicity of 2,4,5-T.

On the basis of our results we think that 2,4,5-T should be placed in the C2 or C3 priority group defined in the Report on Pesticides by the U.S. Department of Health (1969). The C group comprises chemical substances whose activity has been insufficiently assessed. C2 and C3 are the priority groups requiring additional data.

The classification of 2,4,5-T in the C2 or C3 groups implies that further testing in greater numbers of animals and in other species such as the rat and the dog is necessary. Until these results are available this chemical should be handled with caution.

We are highly indebted to Dr V. K. Rowe, Director of Toxicological Affairs, Health and Environmental Research of Dow Chemical U.S.A. for the analysis of 2,4,5-T used in our experiments, to Dr G. Saint-Ruff, maître de recherches Centre Délépine du C.N.R.S., Orléans, who synthesized the 2,4,5-T, and to Mrs F. Apiou for excellent technical assistance. We also wish to thank Dr Gaudebout for helpful advice and Mrs C. Lipcey for revision of the English text.

### REFERENCES

CONNEY, A. H. & LEVIN, W. (1974) Carcinogen Metabolism in Experimental Animals and Man. In *Chemical Carcinogenesis Essays*. W.H.O.–IARC Scientific Publications No. 10, Ed. R. Montesano and L. Tomatis. Lyon: IARC. p. 3.

COURTNEY, K. D., GAYLOR, D. W., HOGAN, M. D., FALK, H. L., BATES, R. P. & MITCHELL, I. (1970) Teratogenic Evaluation of 2,4,5-T. *Science*, **168**, 864.

DRILL, V. A. & HIRATZKA, T. (1953) Toxicity of 2,4-Dichlorophenoxyacetic Acid and 2,4,5-Trichlorophenoxyacetic Acid. A Report on Their Acute and Chronic Toxicity in Dogs. *Arch. ind. Hyg. and occup. Med.*, **7**, 61.

EMERSON, J. L., THOMPSON, D. J., STREGING, R. J., GERBIG, C. G. & ROBINSON, V. B. (1971) Teratogenic Studies on 2,4,5-Trichlorophenoxyacetic Acid in the Rat and Rabbit. *Food Cosmet. Toxicol.*, **9**, 395.

FAHRIG, R. (1974) Comparative Mutagenicity Studies with Pesticides. In *Chemical Carcinogenesis Essays*. W.H.O.–IARC Scientific Publications No. 10, Ed. R. Montesano and L. Tomatis. Lyon: IARC. p. 161.

GEHRING, P. J., KRAMER, C. G., SCHWETZ, B. A., ROSE, J. O. & ROWE, V. K. (1973) The Fate of 2,4,5-Trichlorophenoxyacetic Acid (2,4,5-T) Following Oral Administration to Man. *Toxicol. Appl. Pharmacol.*, **26**, 352.

GRIGSBY, B. H. & FARWELL, E. D. (1950) Some Effects of Herbicides on Pasture and Grazing Livestock. *Mich. Agr. Exp. Sta. Quart. Bull.*, **32**, 378.

INNES, J. R. M., ULLAND, B. M., VALERIO, M. G., PETRUCELLI, L., FISHBEIN, L., HART, E. R., PALLOTTA, A. J., BATES, R. R., FALK, H. L., GART, J. J., KLEIN, M., MITCHELL, I. & PETERS, J. (1969) Bioassay of Pesticides and Industrial Chemicals for Tumorigenicity in Mice: A Preliminary Note. *J. nat. Cancer Inst.*, **42**, 1101.

MOORE, J. A., GUPTA, B. N., ZINKL, J. G. & VOS, J. G. (1973) Post-natal Effects of Maternal Exposure to 2,3,7,8-tetrachlorodibenzo-p-dioxin (TCDD). *Environ. Health Perspect.*, **5**, 81.

PETO, R. (1974) Guidelines on the Analysis of Tumour Rates and Death Rates in Experimental Animals. *Br. J. Cancer*, **29**, 101.

PIPER, W. N., ROSE, J. Q., LENG, M. L. & GEHRING, P. J. (1973) The Fate of 2,4,5-Trichlorophenoxyacetic Acid (2,4,5-T) Following Oral Administration to Rats and Dogs. *Toxicol. Appl. Pharmacol.*, **26**, 339.

REPORT of the Secretary's Commission on Pesticides and Their Relationship to Environmental Health (1969) U.S. Dept. of Health, Education and Welfare.

ROWE, V. K. & HYMAS, T. A. (1954) Summary of Toxicological Information on 2,4-D and 2,4,5-T Type Herbicides and an Evaluation of the Hazards to Livestock Associated with Their Use. *Amer. J. Vet. Res.*, **15**, 622.

SCHWETZ, B. A., NORRIS, J. M., SPARSCHU, G. L., ROWE, V. K., GEHRING, P. J., EMERSON J. L., & GERBIG, C. G. (1973) Toxicology of Chlorinated Dibenzo-p-dioxins. *Environ. Health Perspect.*, **No. 5**, p. 87.

SPARSCHU, G. L., DUNN, F. L., LISOWE, R. W. & ROWE, V. K. (1971) Study on the Effects of High Levels of 2,4,5-Trichlorophenoxyacetic Acid on Foetal Development in the Rat. *Food Cosmet. Toxicol.*, **9**, 527.

TOMATIS, L. & MOHR, U. (1973) Transplacental Carcinogenesis. W.H.O.–IARC Scientific Publications **No. 4**. Lyon: IARC.

TOTH, B. (1968) A Critical Review of Experiments in Chemical Carcinogenesis Using Newborn Animals. *Cancer Res.*, **28**, 727.

TUNG, TON THAT (1973) Le Cancer Primaire du Foie au Viet-Nam. *Chirurgie*, **99**, 427.


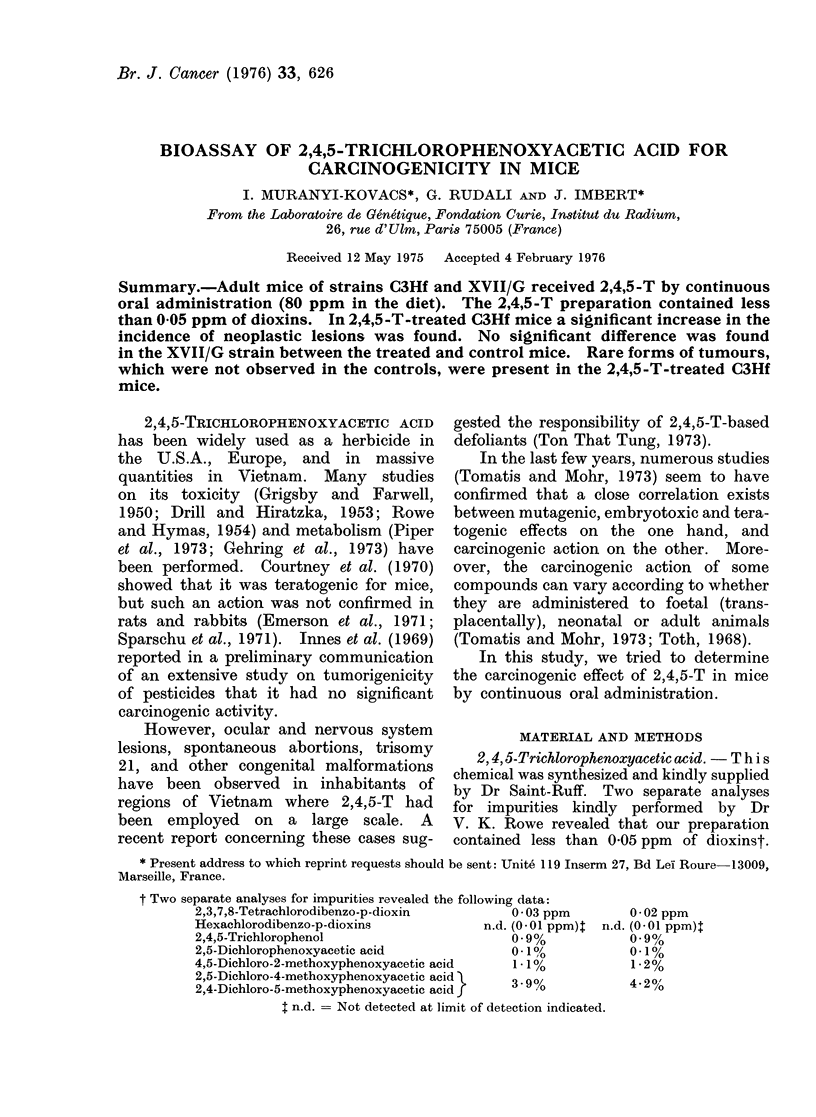

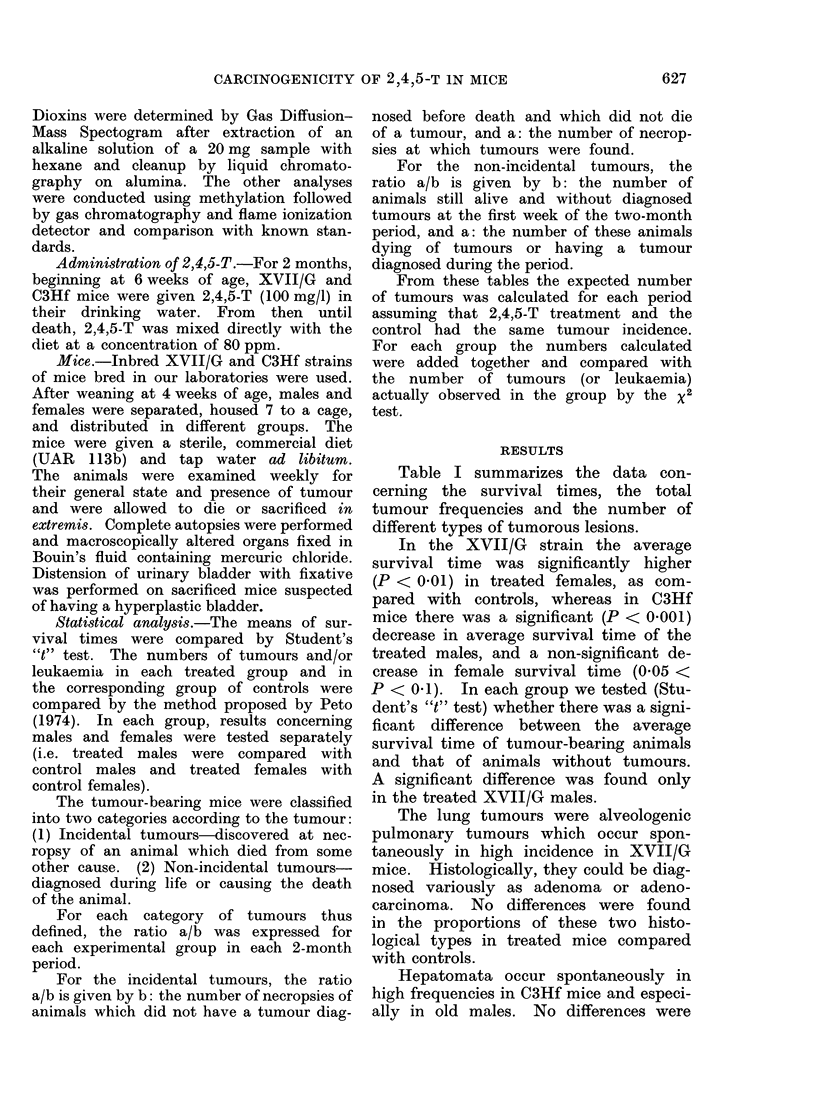

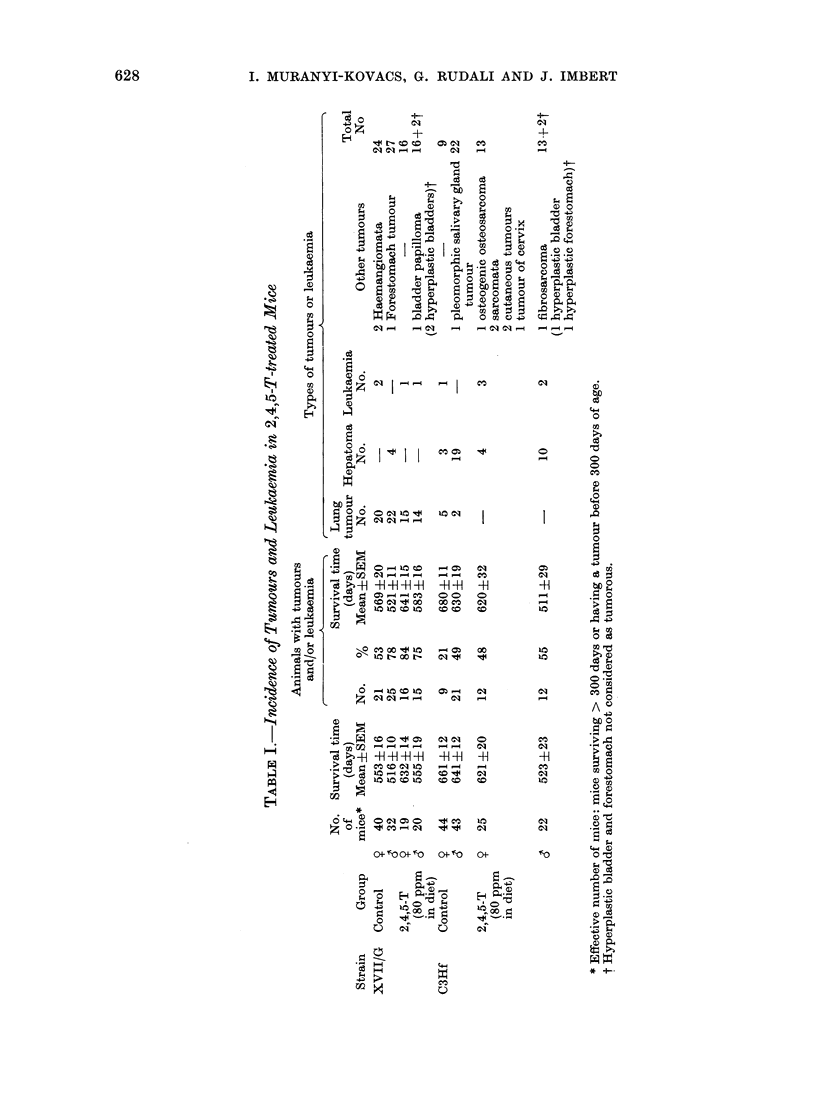

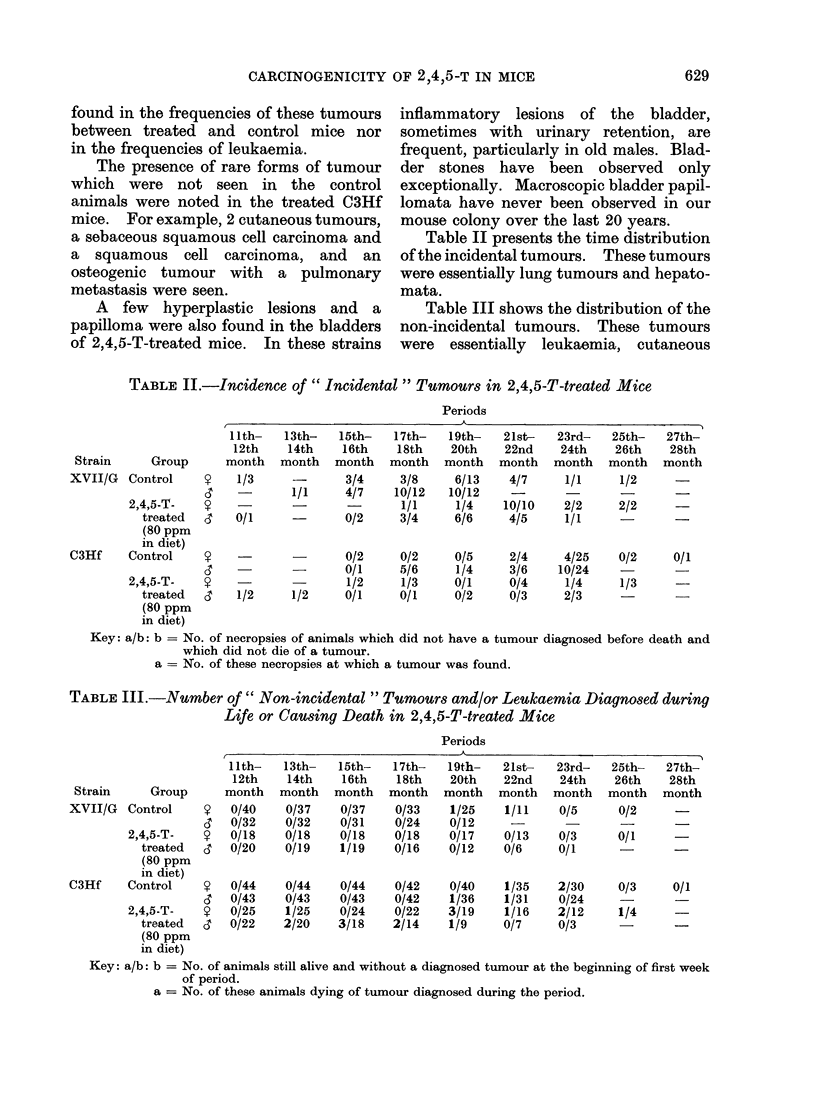

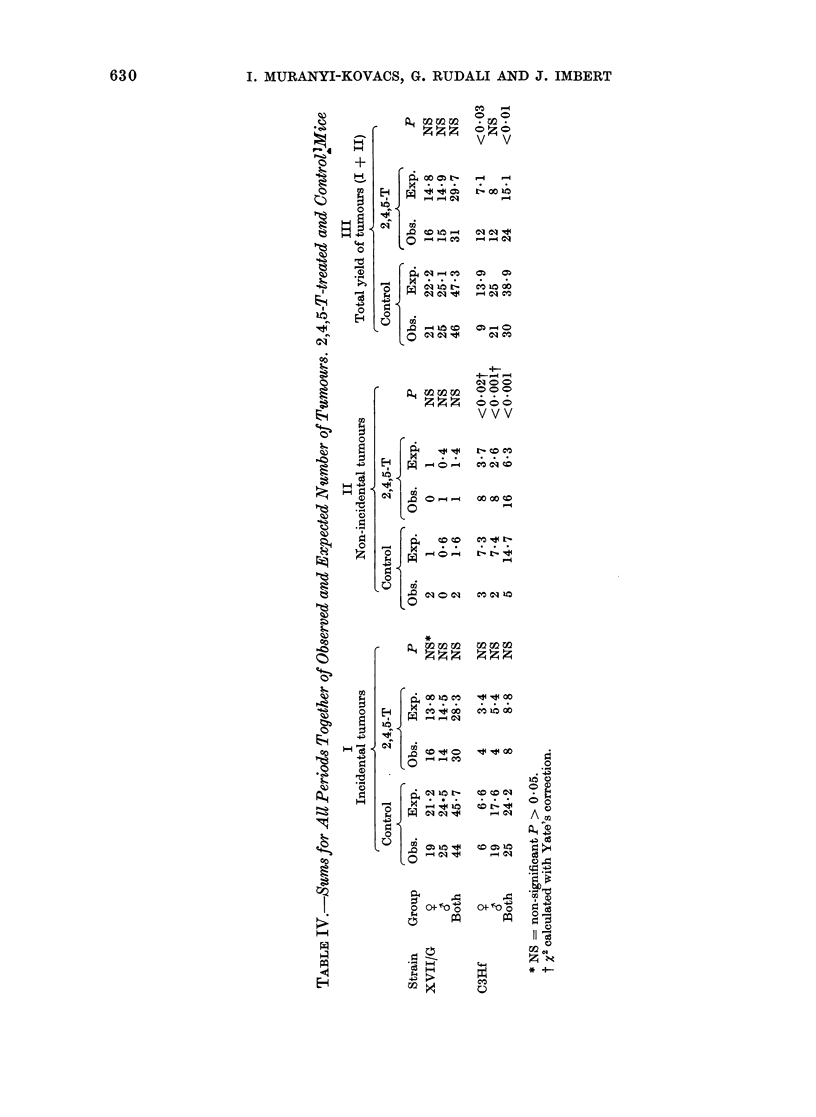

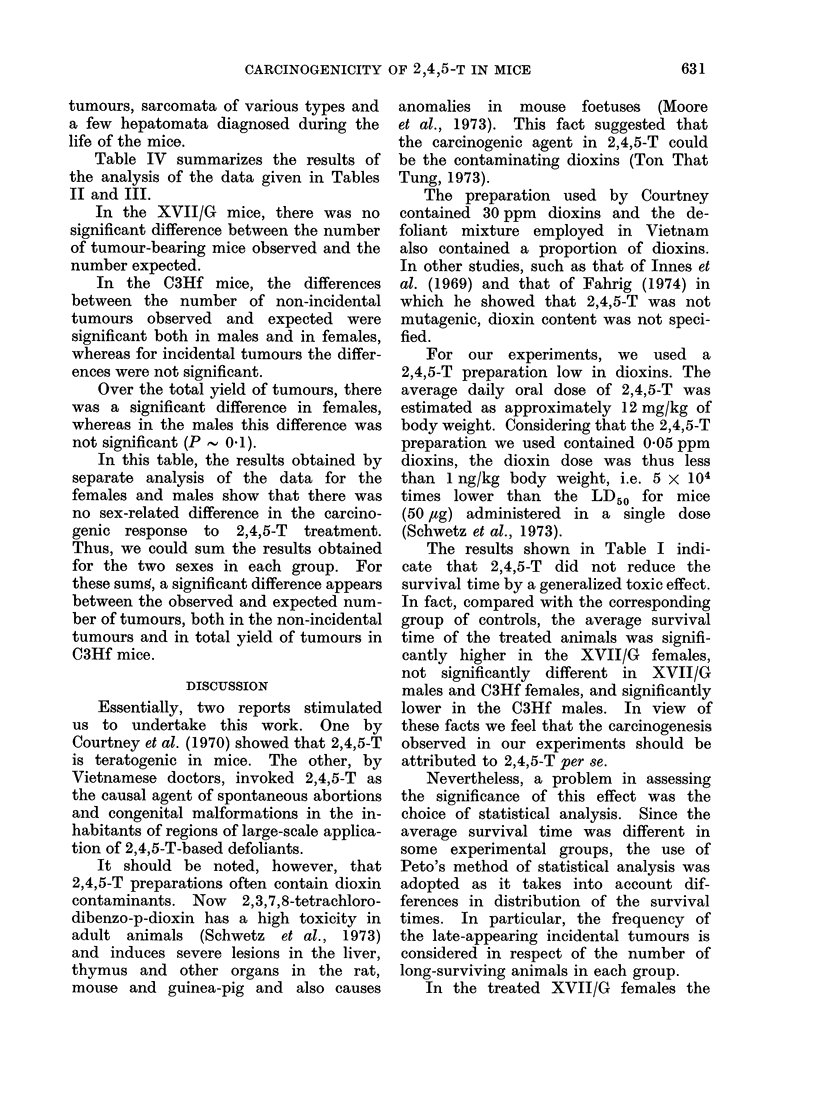

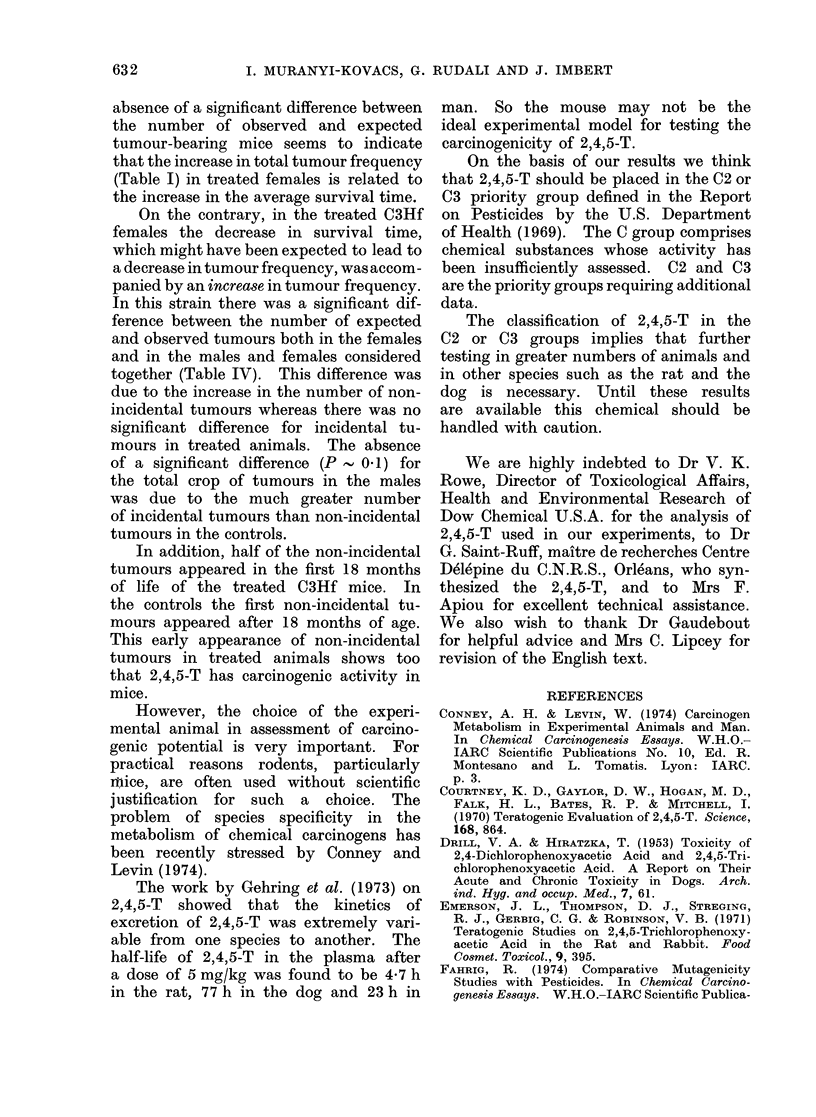

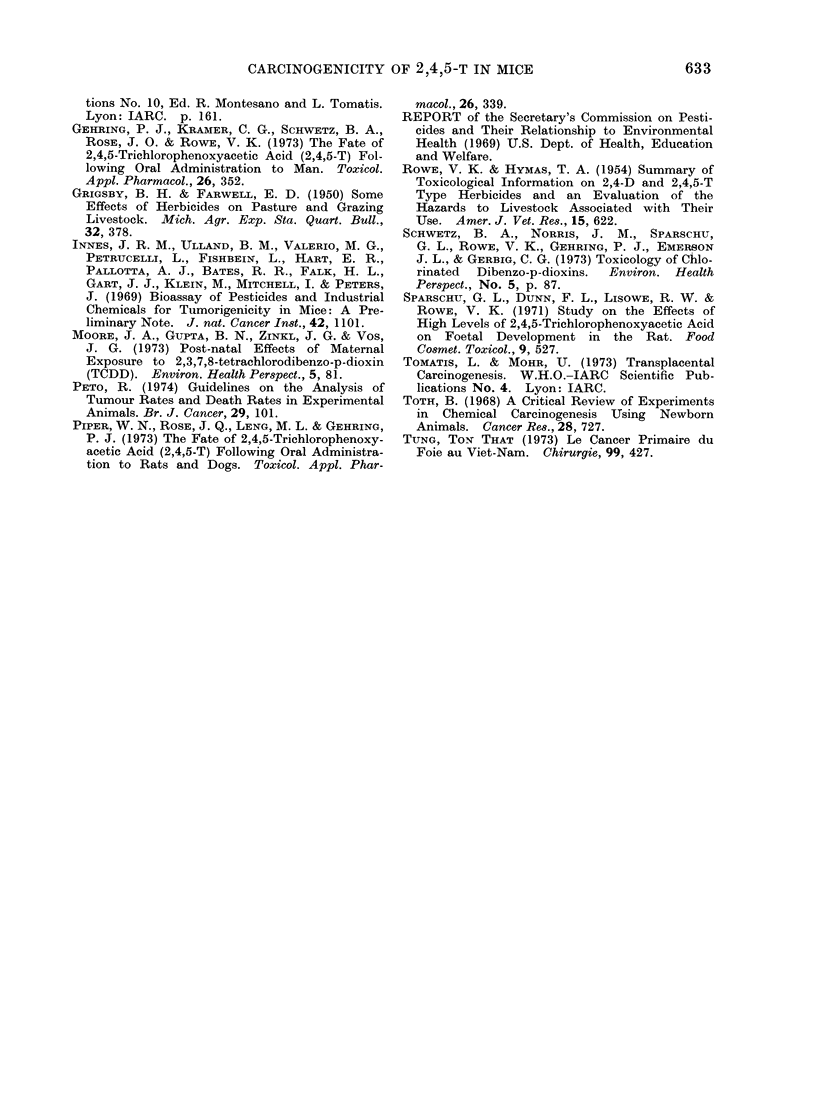


## References

[OCR_00858] Courtney K. D., Gaylor D. W., Hogan M. D., Falk H. L., Bates R. R., Mitchell I. (1970). Teratogenic evaluation of 2,4,5-T.. Science.

[OCR_00864] DRILL V. A., HIRATZKA T. (1953). Toxicity of 2,4-dichlorophenoxyacetic acid and 2,4,5-trichlorophenoxyacetic acid; a report on their acute and chronic toxicity in dogs.. AMA Arch Ind Hyg Occup Med.

[OCR_00871] Emerson J. L., Thompson D. J., Strebing R. J., Gerbig C. G., Robinson V. B. (1971). Teratogenic studies on 2,4,5-trichlorophenoxyacetic acid in the rat and rabbit.. Food Cosmet Toxicol.

[OCR_00888] Gehring P. J., Kramer C. G., Schwetz B. A., Rose J. Q., Rowe V. K. (1973). The fate of 2,4,5-trichlorophenoxyacetic acid (2,4,5-T) following oral administration to man.. Toxicol Appl Pharmacol.

[OCR_00910] Moore J. A., Gupta B. N., Zinkl J. G., Vos J. G. (1973). Postnatal effects of maternal exposure to 2,3,7,8-tetrachlorodibenzo-p-dioxin (TCDD).. Environ Health Perspect.

[OCR_00916] Peto R. (1974). Editorial: Guidelines on the analysis of tumour rates and death rates in experimental animals.. Br J Cancer.

[OCR_00921] Piper W. N., Rose J. Q., Leng M. L., Gehring P. J. (1973). The fate of 2,4,5-trichlorophenoxyacetic acid (2,4,5-T) following oral administration to rats and dogs.. Toxicol Appl Pharmacol.

[OCR_00935] ROWE V. K., HYMAS T. A. (1954). Summary of toxicological information on 2,4-D and 2,4,5-T type herbicides and an evaluation of the hazards to livestock associated with their use.. Am J Vet Res.

[OCR_00949] Sparschu G. L., Dunn F. L., Lisowe R. W., Rowe V. K. (1971). Study of the effects of high levels of 2,4,5-trichlorophenoxyacetic acid on foetal development in the rat.. Food Cosmet Toxicol.

[OCR_00966] Ton-That-Tung (1973). Le cancer primaire du foie au Viêt-Nam.. Chirurgie.

[OCR_00961] Toth B. (1968). A critical review of experiments in chemical carcinogensis using newborn animals.. Cancer Res.

